# Whole Genome Sequence and Phylogenetic Analysis Show *Helicobacter pylori* Strains from Latin America Have Followed a Unique Evolution Pathway

**DOI:** 10.3389/fcimb.2017.00050

**Published:** 2017-02-28

**Authors:** Zilia Y. Muñoz-Ramírez, Alfonso Mendez-Tenorio, Ikuko Kato, Maria M. Bravo, Cosmeri Rizzato, Kaisa Thorell, Roberto Torres, Francisco Aviles-Jimenez, Margarita Camorlinga, Federico Canzian, Javier Torres

**Affiliations:** ^1^Laboratorio de Biotecnología y Bioinformática Genómica, ENCB, Instituto Politécnico NacionalCiudad de México, Mexico; ^2^Department of Oncology and of Pathology, Wayne State University School of MedicineDetroit, MI, USA; ^3^Grupo de Investigación en Biología del Cáncer, Instituto Nacional de CancerologiaBogota, Colombia; ^4^Dipartmento di Ricerca Traslazionale e Nuove Tecnologie in Medicina e Chirurgia, Università di PisaPisa, Italy; ^5^Department of Microbiology, Tumor and Cell Biology, Karolinska InstitutetStockholm, Sweden; ^6^Unidad de Investigacion en Enfermedades Infecciosas, IMSSCiudad de México, Mexico; ^7^Genomic Epidemiology Group, German Cancer Research Center (DKFZ)Heidelberg, Germany

**Keywords:** *H. pylori*, phylogenetic analysis, whole genome sequence, Latin America

## Abstract

*Helicobacter pylori* (HP) genetics may determine its clinical outcomes. Despite high prevalence of HP infection in Latin America (LA), there have been no phylogenetic studies in the region. We aimed to understand the structure of HP populations in LA mestizo individuals, where gastric cancer incidence remains high. The genome of 107 HP strains from Mexico, Nicaragua and Colombia were analyzed with 59 publicly available worldwide genomes. To study bacterial relationship on whole genome level we propose a virtual hybridization technique using thousands of high-entropy 13 bp DNA probes to generate fingerprints. Phylogenetic virtual genome fingerprint (VGF) was compared with Multi Locus Sequence Analysis (MLST) and with phylogenetic analyses of *cagPAI* virulence island sequences. With MLST some Nicaraguan and Mexican strains clustered close to Africa isolates, whereas European isolates were spread without clustering and intermingled with LA isolates. VGF analysis resulted in increased resolution of populations, separating European from LA strains. Furthermore, clusters with exclusively Colombian, Mexican, or Nicaraguan strains were observed, where the Colombian cluster separated from Europe, Asia, and Africa, while Nicaraguan and Mexican clades grouped close to Africa. In addition, a mixed large LA cluster including Mexican, Colombian, Nicaraguan, Peruvian, and Salvadorian strains was observed; all LA clusters separated from the Amerind clade. With *cagPAI* sequence analyses LA clades clearly separated from Europe, Asia and Amerind, and Colombian strains formed a single cluster. A NeighborNet analyses suggested frequent and recent recombination events particularly among LA strains. Results suggests that in the new world, *H. pylori* has evolved to fit mestizo LA populations, already 500 years after the Spanish colonization. This co-adaption may account for regional variability in gastric cancer risk.

## Introduction

*Helicobacter pylori* is a Gram negative bacterium that colonizes the human gastric mucosa of approximately 50% of the human population and induces a chronic inflammatory process. The infection can remain active in gastric mucosa for decades and may progress into peptic ulcers, mucosa associated lymphoid tissue (MALT) lymphoma and gastric cancer (GC). Accordingly, *H. pylori* was classified as a type I carcinogen by the WHO in 1994 (International Agency for Research on Cancer, 1994). The *H. pylori* pathogenic mechanisms are complex, but one of the best recognized virulence factors involved in carcinogenesis is the CagA protein and the presence of the type four secretion system (T4SS) encoded in the cag pathogenicity island (*cagPAI*) (Blaser et al., 1995; Odenbreit et al., 2000; Ohnishi et al., 2008). The molecular syringe encoded by the *cagPAI* is used to inject the CagA oncoprotein into the gastric epithelial cells inducing a pro-inflammatory and antiapoptotic response, affecting cell-to-cell communication by disrupting the tight junctions, and altering cell motility with cytoskeleton re-arrangements (Segal et al., 1996; Brandt et al., 2005; Shibata et al., 2006). Several other genes might be associated with disease development and analysis at whole genome level is necessary to better understand the mechanisms involved in cancer pathogenesis. Currently, the NCBI reports over 500 *H. pylori* genome sequences obtained from strains of different geographical regions around the world, although most of these sequences are not fully assembled. A comprehensive study of the genome of 39 *H. pylori* strains estimated a total of 59,958 genes for the pangenome, and a core or central genome comprised of approximately 1,193 core gene-families (Ali et al., 2015).

*Helicobacter pylori* has infected the human stomach for at least 88,000–116,000 years (Moodley et al., 2012) and co-evolved with our ancestors during the first human migrations from east Africa approximately 60,000 years ago (Linz et al., 2007). The study of *H. pylori* population genetics has been of great interest because of its clinical and phylogeographic significance. One characteristic that distinguishes *H. pylori* from other human bacterial pathogens is the extensive genetic diversity among isolates due to point mutations and high recombination rates (Suerbaum et al., 1998; Achtman et al., 1999; Björkholm et al., 2001; Falush et al., 2001). Earlier studies on the structure of *H. pylori* populations employed the Multi Locus Sequence Typing (MLST) based on seven housekeeping genes, and identified six main bacterial groups: hpAfrica1, hpAfrica2, hpEastAsia, hpAsia2, hpEurope and hpSahul; and other more recent subpopulations: hpEastAsia was divided into the hspAmerind, hspEAsia, and hspMaori, and hpAfrica1 into hspWAfrica and hspSAfrica (Falush et al., 2003; Moodley et al., 2009). Studies in native, genetically isolated communities in regions of Latin America (LA) have shown that in Amerindian groups the Asian ancestry remains conserved millennia after their migration to the Americas through the Bering Strait (Kersulyte et al., 2010, 2015; Camorlinga-Ponce et al., 2011). However, a few studies have analyzed *H. pylori* of LA mestizo populations (individuals with a combination of European, African, and Amerind ancestry). These studies have suggested that LA groups have an admixture of European and African ancestry, a result of the Spanish conquest and the migration of African slaves (Falush et al., 2003; Thorell et al., 2016).

Current phylogenetic classification of most bacteria is based on the 16S rRNA gene, although this molecule is not able to distinguish between closely related organisms as in the case of strains or subspecies. Although, phylogeny have been improved by increasing the number of genes analyzed like MLST, the evolution of one gene hardly describes the evolution of whole organisms. Analyses of a limited number of genes cannot uncover more complex evolutionary events. However, current algorithms for whole genome comparison are computer-intensive, and the development of specialized computer applications for efficiently exploring genome information remains a challenge.

In this work we aimed to study the structure of *H. pylori* population in regions of North, Central and South LA. To this end, we analyzed the whole genome of *H. pylori* isolates from Mexico, from Nicaragua and from Colombia, and performed phylogenetic analysis including also other publicly available worldwide genomes. We propose an approach for genome comparison by means of genomic fingerprints estimated by a virtual hybridization technique using thousands of randomly generated small-size DNA probes. This technique was compared with the well-known MLST technique and with the sequence analyses of the *cagPAI* virulence island. We found that whole genome fingerprints improved the definition of *H. pylori* populations, separating European and Asian from LA strains. Furthermore, clades with exclusively Colombian, Mexican, or Nicaraguan strains were observed, suggesting that *H. pylori* has evolved to fit LA mestizo populations, already 500 years after the Spanish colonization, probably to reduce disease risk.

## Materials and methods

### Clinical isolates

*Helicobacter pylori* strains were isolated from individuals diagnosed with different gastric pathologies in Mexico and Colombia. Stomach biopsy samples were homogenized and processed as follow, in Mexico homogenates were inoculated on Blood agar base (Becton Dickinson, New Jersey, USA), with Campylobacter-selective antibiotics supplement (Oxoid, LTD. England) and with 5% sheep blood; whereas in Colombia homogenates were grown on blood agar plates, supplemented with 7% horse serum (Invitrogen), 1% Vitox (Oxoid), and Campylobacter-selective supplement (Oxoid). In both places agar plates were incubated at 37°C under a 10% CO2 atmosphere and genomic DNA was extracted using the DNeasy Mini Kit (Qiagen, Hilden Germany). The Nicaraguan strains were isolated and sequenced in a previously published project and a detailed description can be found in Thorell et al. (2016). Patients studied in Mexico were from the central region of the country, where the mestizo represents over 90% of the population (Silva-Zolezzi et al., 2009). For Nicaragua, the subjects sampled in this cohort were mainly from Managua and surrounding areas and are consequently likely to be of mostly mestizo origin^1^. Population sampled in Colombia was from the Andean subregion Central East, where mestizo represents over 90% of the population (Ossa et al., 2016).

Each DNA sample was processed to prepare a genomic library and submitted to whole genome sequencing with the HiSeq 2000 platform (Illumina) using the paired-end method. In total, this resulted in a collection of 107 draft genomes from three LA countries (Colombia, Mexico, and Nicaragua; Table 1). The clinical studies where patients were originally recruited were approved by the respective Institutional ethical committee in Mexico, Nicaragua, or Colombia.

**Table 1:** **Description of origin and clinical source of the Latin American genomes analysed in this study**.

**Strain**	**Country**	**Clinical origin**	**Age of patient**
CA22019	Colombia	Atrophic gastritis	38
CA22020	Colombia	Atrophic gastritis	64
CA22095	Colombia	Atrophic gastritis	52
CA22311	Colombia	Atrophic gastritis	47
CA22312	Colombia	Atrophic gastritis	57
CA22327	Colombia	Atrophic gastritis	52
CA22335	Colombia	Atrophic gastritis	ND
CA22337	Colombia	Atrophic gastritis	46
CA22339	Colombia	Atrophic gastritis	ND
CA22362	Colombia	Atrophic gastritis	34
CA22393	Colombia	Atrophic gastritis	62
CA24004	Colombia	Atrophic gastritis	80
CA26024	Colombia	Atrophic gastritis	63
CC22093	Colombia	Gastric cancer	43
CC22402	Colombia	Gastric cancer	55
CC26084	Colombia	Gastric cancer	70
CC26093	Colombia	Gastric cancer	70
CC26100	Colombia	Gastric cancer	70
CG22023	Colombia	Gastritis	64
CG22025	Colombia	Gastritis	36
CG22087	Colombia	Gastritis	30
CG22322	Colombia	Gastritis	44
CG22366	Colombia	Gastritis	38
CG22367	Colombia	Gastritis	30
CG22370	Colombia	Gastritis	37
CG22371	Colombia	Gastritis	37
CG22378	Colombia	Gastritis	43
CG22385	Colombia	Gastritis	59
CG22389	Colombia	Gastritis	63
CM22013	Colombia	Metaplasia	78
CM22021	Colombia	Metaplasia	59
CM22046	Colombia	Metaplasia	64
CM22315	Colombia	Metaplasia	49
CM22331	Colombia	Metaplasia	55
CM22341	Colombia	Metaplasia	47
CM22346	Colombia	Metaplasia	59
CM22347	Colombia	Metaplasia	45
CM22351	Colombia	Metaplasia	43
CM22360	Colombia	Metaplasia	50
CM22368	Colombia	Metaplasia	61
CM22388	Colombia	Metaplasia	58
CM22390	Colombia	Metaplasia	53
MM2003-103	Mexico	Metaplasia	52
MC2006-52	Mexico	Gastric cancer	51
MC2011-145	Mexico	Gastric cancer	34
MCms1054	Mexico	Gastric cancer	68
MCms1055	Mexico	Gastric cancer	78
MCms1063	Mexico	Gastric cancer	60
MCms1078	Mexico	Gastric cancer	34
MCms1080	Mexico	Gastric cancer	58
MCms931	Mexico	Gastric cancer	56
MG2003-107	Mexico	Gastritis	34
MG2003-98	Mexico	Gastritis	51
MG2005-100	Mexico	Gastritis	56
MG2005-98	Mexico	Gastritis	48
MG2006-4	Mexico	Gastritis	39
MG2006-407	Mexico	Gastritis	55
MG2006-479	Mexico	Gastritis	36
MG2011-41	Mexico	Gastritis	69
MGms13	Mexico	Gastritis	42
MGms15	Mexico	Gastritis	66
MGms167	Mexico	Gastritis	36
MGms176	Mexico	Gastritis	44
MGms2	Mexico	Gastritis	47
MGms203	Mexico	Gastritis	34
MGms23	Mexico	Gastritis	34
MGms44	Mexico	Gastritis	66
MM2004-20	Mexico	Metaplasia	49
MM2005-72	Mexico	Metaplasia	52
MM2006-103	Mexico	Metaplasia	72
MM2005-126	Mexico	Metaplasia	52
MM2006-480	Mexico	Metaplasia	58
MM2006-56	Mexico	Metaplasia	73
MM2012-26	Mexico	Metaplasia	68
MU2003-84	Mexico	Metaplasia	46
MU2004-2	Mexico	Metaplasia	41
Nic01_A	Nicaragua	Atrophic Gastritis	48
Nic02_A	Nicaragua	Atrophic Gastritis	66
Nic03_A	Nicaragua	Atrophic Gastritis	29
Nic04_A	Nicaragua	Atrophic Gastritis	34
Nic05_A	Nicaragua	Atrophic Gastritis	27
Nic06_A	Nicaragua	Atrophic Gastritis	47
Nic07_A	Nicaragua	Atrophic Gastritis	27
Nic08_C	Nicaragua	Gastritis	27
Nic09_A	Nicaragua	Atrophic Gastritis	53
Nic10_A	Nicaragua	Gastritis	35
Nic11_A	Nicaragua	Atrophic Gastritis	58
Nic12_A	Nicaragua	Atrophic Gastritis	32
Nic13_A	Nicaragua	Gastritis	30
Nic14_A	Nicaragua	Gastritis	36
Nic15_A	Nicaragua	Gastritis	44
Nic16_A	Nicaragua	Gastritis	24
Nic17_A	Nicaragua	Atrophic Gastritis	35
Nic18_A	Nicaragua	Atrophic Gastritis	27
Nic19_A	Nicaragua	Metaplasia	47
Nic20_A	Nicaragua	Metaplasia	47
Nic21_A	Nicaragua	Metaplasia	53
Nic22_A	Nicaragua	Gastritis	26
Nic23_A	Nicaragua	Metaplasia	55
Nic25_A	Nicaragua	Metaplasia	58
Nic26_A	Nicaragua	Gastritis	58
Nic27_A	Nicaragua	Gastritis	40
Nic28_A	Nicaragua	Gastritis	18
Nic29_A	Nicaragua	N/A	60
Nic30_A	Nicaragua	Gastritis	53
Nic31_A	Nicaragua	N/A	30
Nic32_A	Nicaragua	Gastritis	24

*Sequences and clinical information of Nicaraguan strains are deposited at Sequence Read Archive, SRA with the accession number PRJNA242766*.

### Selection of *H. pylori* genomes from strains available at NCBI database

To analyze the phylogeny of the LA isolates, we downloaded the genome sequence of 59 *H. pylori* strains from other regions of the world, available in the NCBI database (Table S1). Initially, we selected only strains whose genome were fully assembled into one single sequence, and with a clear statement concerning geographic origin. Besides those, we also incorporated two other strains with incomplete draft genomes (Iceman and Sahul64 strains), due to their historical and geographical relevance. We used these files to do phylogeographic analyses of their whole genomes, of their *cagPAI* and *cagA* sequence and to perform MLST. In addition, for the whole genome analyses (VGF, see below) we downloaded the genome of *H. acinonychis* to use it as reference to root the phylogenetic trees.

### Virtual genome fingerprint

For this analysis the bacterial genome sequences provided in FASTA format were used to determine the Virtual Genome Fingerprints (VGF) using the VAMPhyRE software (Mendez-Tenorio et al., manuscript in preparation). Draft sequences were provided as a single string of concatenated contigs or scaffold sequences. The analysis consisted of two main stages; the first consisted in calculation of VGF using a collection of 15,264 highly diverse 13-mer *probe* sequences (VAMPhyRe Probe Set, VPS). The probe collection was tested with the entire sequence of both complete and draft genomes (including all concatenated contigs) using virtual hybridization, and allowing only one mismatch in both (+ and −) genome strands, in order to find all the complementary sites for the VPS. The detailed list of the sites identified by the virtual hybridization approach in a given genome is known as Virtual Genomic Fingerprint (VGF), and is characteristic for each bacterial genome. In the second stage the genomic distances were estimated by comparing the VGF of each bacterial genome in order to determine the number of sites shared by all pair of genomic fingerprints. Since some sites shared between genomes may correspond to non-homologous positions, the sites were extended by three positions to both left to right sites, for a total of 19 nt length. A site was considered as homologous between two genomes if the number of matches between the two sequences was ≥16 out of 19; a previous statistical analysis with unrelated sequences showed that by using such values no shared signals were observed. From the number of shared homologous signals between a pair of sequences, a similarity coefficient and a distance value for each pair of genomic fingerprints are estimated using an approach previously described (Nei and Li, 1979). This method was used to build a matrix of distances for all pairs of genomic fingerprints. Virtual Genomic Fingerprints can be calculated from both draft and complete genomes. Additionally, VGFs for *H. pylori* without the *cagPAI* island where calculated by subtracting the VGF of the island from the VGF of the whole genome. The matrix of distances calculated with VAMPhyRe was used to build phylogenomic trees using MEGA5.2.2 (Tamura et al., 2011). Additionally, Minimal Spanning and Split Decomposition phylogenomic networks (Huson and Bryant, 2006) were also calculated from the matrix of distances using SplitsTree4.

For the MLST analyses we selected the 7 *H. pylori* house-keeping genes previously described (Achtman et al., 1999) in the 110 Latin American genomes as well as in the 61 NCBI available genomes selected for this study (Table S1).

### Phylogenetic analyses of *cagPAI* and MLST genes

We first selected the *cagPAI* and MLST genes from 10 NCBI reference strains and aligned them by “reverse translation” (Wernersson and Pedersen, 2003) with MEGA 5.02. Next, the individual gene alignments were used to calculate Nucleotide Hidden Markov Models (NHMMs) with hmmbuild from the HMMER 3.1 software (Wheeler and Eddy, 2013). Then, we searched for these NHMMs on the complete and draft genomes used in this study with the nhmmer software, and the most significant gene alignments were selected, extracted and the reading frame of each gene verified, aligned by reverse translation with MEGA 5.02 and concatenated. The concatenated alignments were used for phylogenetic analysis of the *cagPAI* and MLST genes with MEGA 5.02. Distance-based phylogenetic trees for the *cagPAI* and MSLT genes were calculated using the T92+G+I (Tamura model with Gamma function and Invariable sites). Bootstrap analysis was performed with 1,000 replications, and Phylogenetic/Phylogenomic trees were edited and annotated with iTol (Interactive Tree Of Life) v3.

### NeighborNet analyses to study interaction within strains

To better document traces of previous interactions with ancestors we build the NeighborNet. For this, distance matrices obtained with the VGF analyses were converted to Nexus format and used as input file to generate phylogenetic networks using SplitsTree v4.14.2 software (Huson and Bryant, 2006). The network was computed choosing the Ordinary Least Squares Variance and the “Equal Angles” Split Transformation parameters.

## Results

### Distribution of Latin American *H. pylori* populations using classical MLST analysis

**Figure 1:**
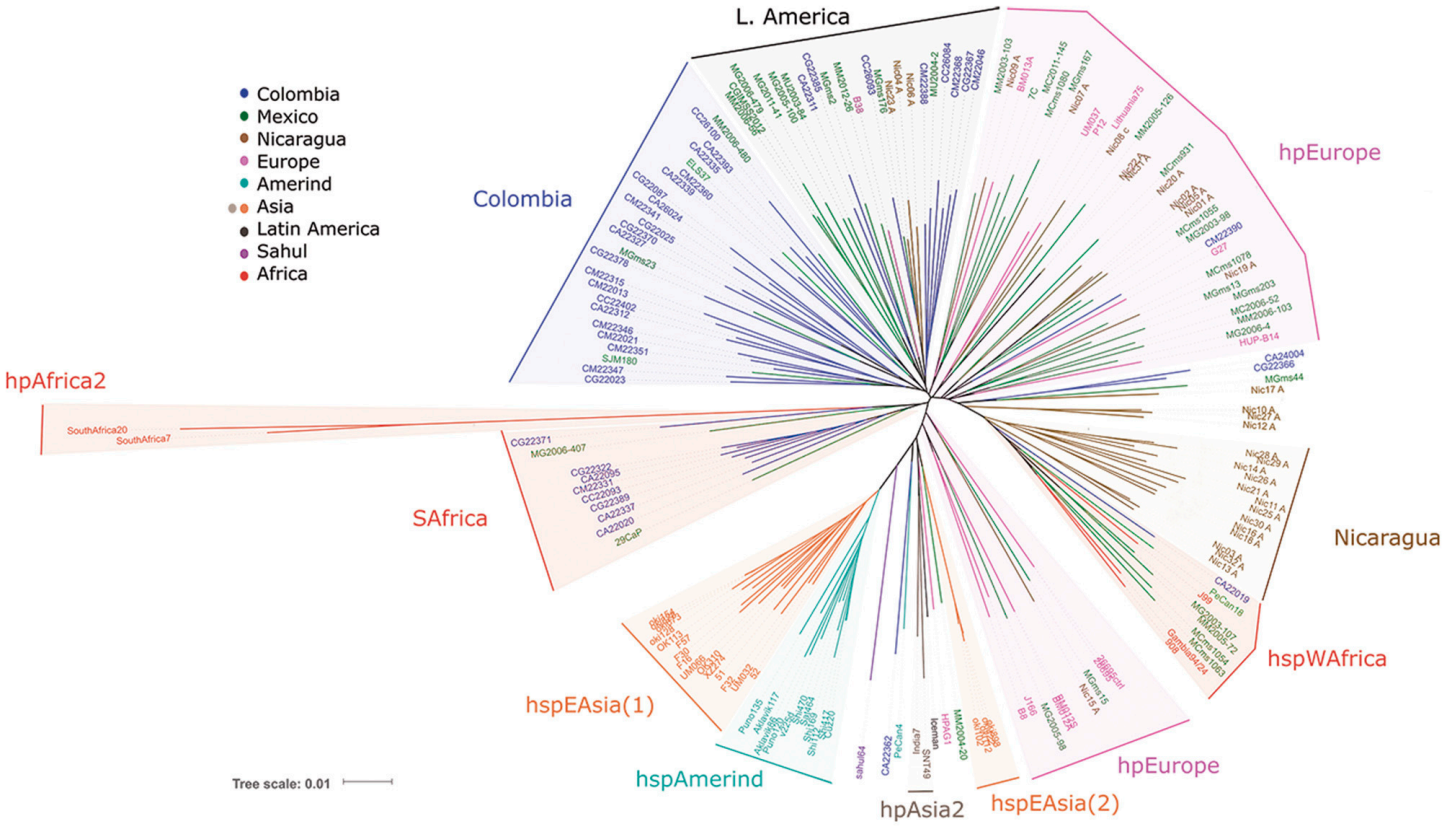
**Phylogenetic analyses of 113 Latin American and 54 worldwide *H. pylori* strains using the Multi Locus Sequence Typing method with seven housekeeping genes (Falush et al., 2003)**.

The initial analyses by MLST (Figure 1) showed the well-described clusters of East Asian *H. pylori*, including the subpopulations hspEAsia and hspAmerind, consistent with previous reports (Domínguez-Bello et al., 2008; Kersulyte et al., 2010); it also showed the hspWAfrica population where several Nicaraguan and some Mexican strains clustered. The hpAfrica2 group clearly clustered distant to all other populations, which is in agreement with published work (Linz et al., 2007). Interestingly, for a number of Colombian and a few Mexican strains their closest common ancestor was this hpAfrica2 group (SAfrica group, Figure 1). European strains were spread into several clusters intermingled with Mexican, Colombian and Nicaraguan strains (hpEurope, Figure 1). Still, besides this dispersed distribution of European and LA strains, one separate cluster contained exclusively LA isolates, except for B38, an isolate from France (LAmerica group, Figure 1). In addition, there was another cluster formed mostly with Colombian isolates (Colombia group, Figure 1), which also included a few other LA isolates from Mexico, El Salvador, and Peru. Thus, already with MLST some separation of LA clusters was evident. With MLST the recently described *Iceman* strain grouped within the hpAsia2 cluster, as reported (Maixner et al., 2016).

### Virtual genomic fingerprinting (VGF) results in a better separation of Latin American *H. pylori* subpopulations

**Figure 2:**
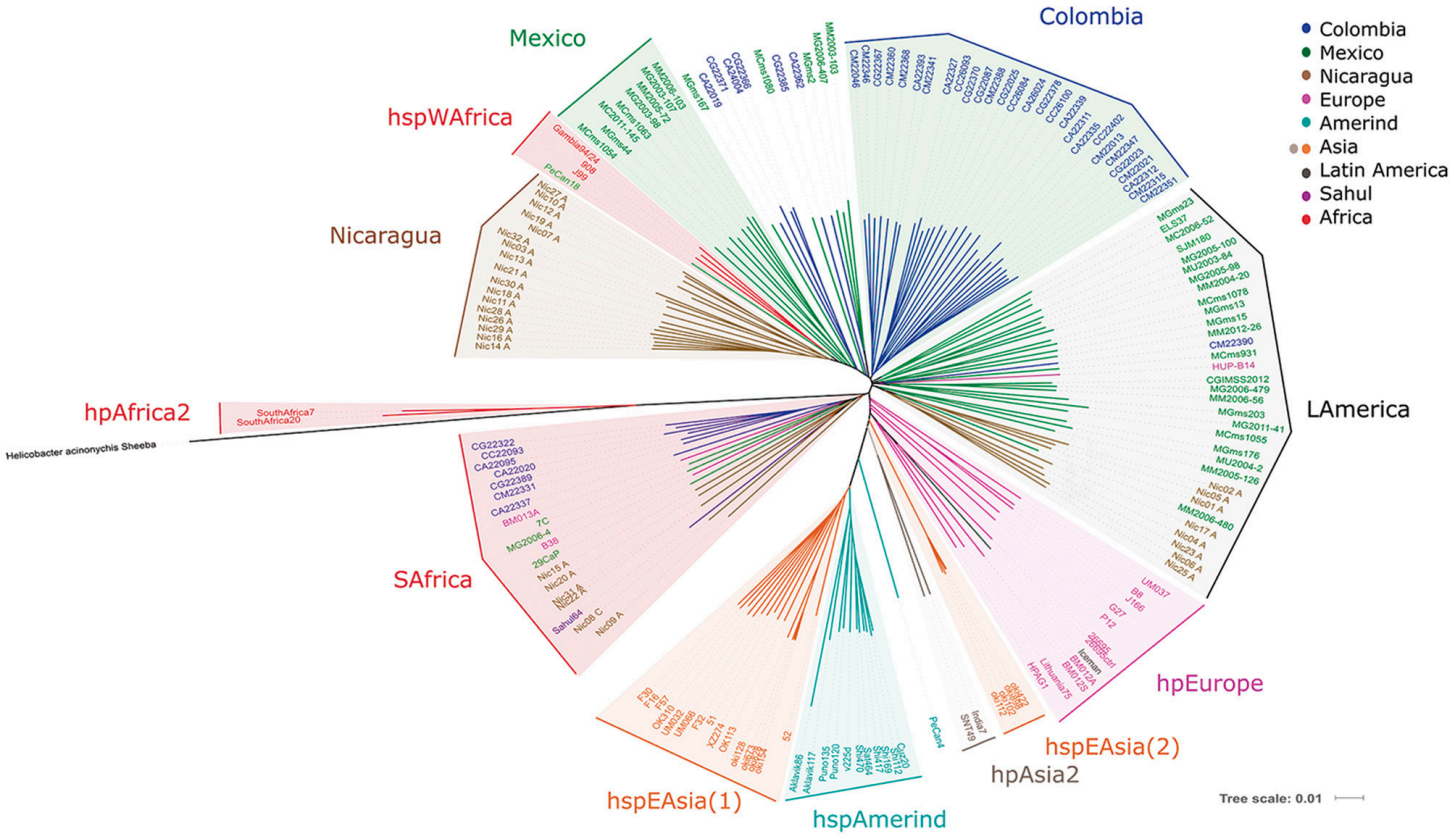
**Phylogenetic analyses of 113 Latin American and 54 worldwide *H. pylori* strains using whole genome sequences analyzed with a virtual hybridization technique (VGF)**. The genome of the related *H. acinonychis* Sheeba strain was used to root the tree.

The analyses of whole genome sequences with our proposed virtual hybridization technique yielded results partially consistent with MLST; thus, hpAfrica2 strains were the most distant group from the rest of the populations, confirming their position as the root of the tree; in fact, this group was closer to the *H. acinonychis* species than any other *H. pylori* group, as expected (Figure 2). The tree also confirmed the formation of the hpEastAsia population with the subpopulations of hspEAsia split in 2 [hspEAsia(1) and (2)], and the hspAmerind including North and South American Amerind isolates. However, in contrast with MLST, the European isolates clustered in a better defined separate group and did not intermingle with LA strains (Figure 2). With VGF the *Iceman* strain clustered with hpEurope and not with hpAsia2 as observed with MLST.

For the LA strains, VGF revealed markedly different patterns compared to MLST. In particular, we observed that most of the Colombian isolates clustered in a separate group (Colombia group, Figure 2), which included a larger number of Colombia strains than observed in the Colombia group with MLST (Figure 1). Another cluster formed with isolates of Mexican origin (Mexico group), and a third formed exclusively with Nicaraguan strains (Nicaragua group). Whereas the Colombian cluster was clearly separated from hpEurope, hspEAsia, hspAmerind, and hspWAfrica, the Nicaragua and Mexico clusters grouped close to hspWAfrica (Figure 2). Of note, a large group comprised of LA strains from Mexico, Nicaragua, Colombia, Peru, and El Salvador clustered in a mixed LA group (LAmerica group), including also the HUP-B14 Spanish isolate. It should be noted that LA strains were closer to hspWAfrica or hpEurope than to their hspAmerind ancestors. One exception was PeCan4, a strain from Peru that located between hspAmerind and the Indian hpAsia2 isolates.

In addition, a cluster with Colombian and a few Mexican, Nicaraguan, European and Sahul strains grouped close to the hpAfrica2 isolates (SAfrica group, Figure 2); this cluster included most strains that also clustered in the SAfrica group with MLST, but with additional isolates from Nicaragua, Europe (the French B38 and Australian BM013A), and Sahul54. Interestingly, all strains of this group lacked the *cagPAI* island, regardless of their origin, except for one Nicaraguan isolate (Nic20A). Therefore, we repeated the VGF analyses excluding the *cagPAI* sequences. This resulted in very different patterns (Figure S1). The SAfrica group was no longer formed and those members, including the LA as well as the European and Sahul strains now clustered with hpEurope, whereas the distribution of all other strains remained unchanged.

### The analyses of *cagPAI* genes sequences also reveal LA subpopulations

**Figure 3:**
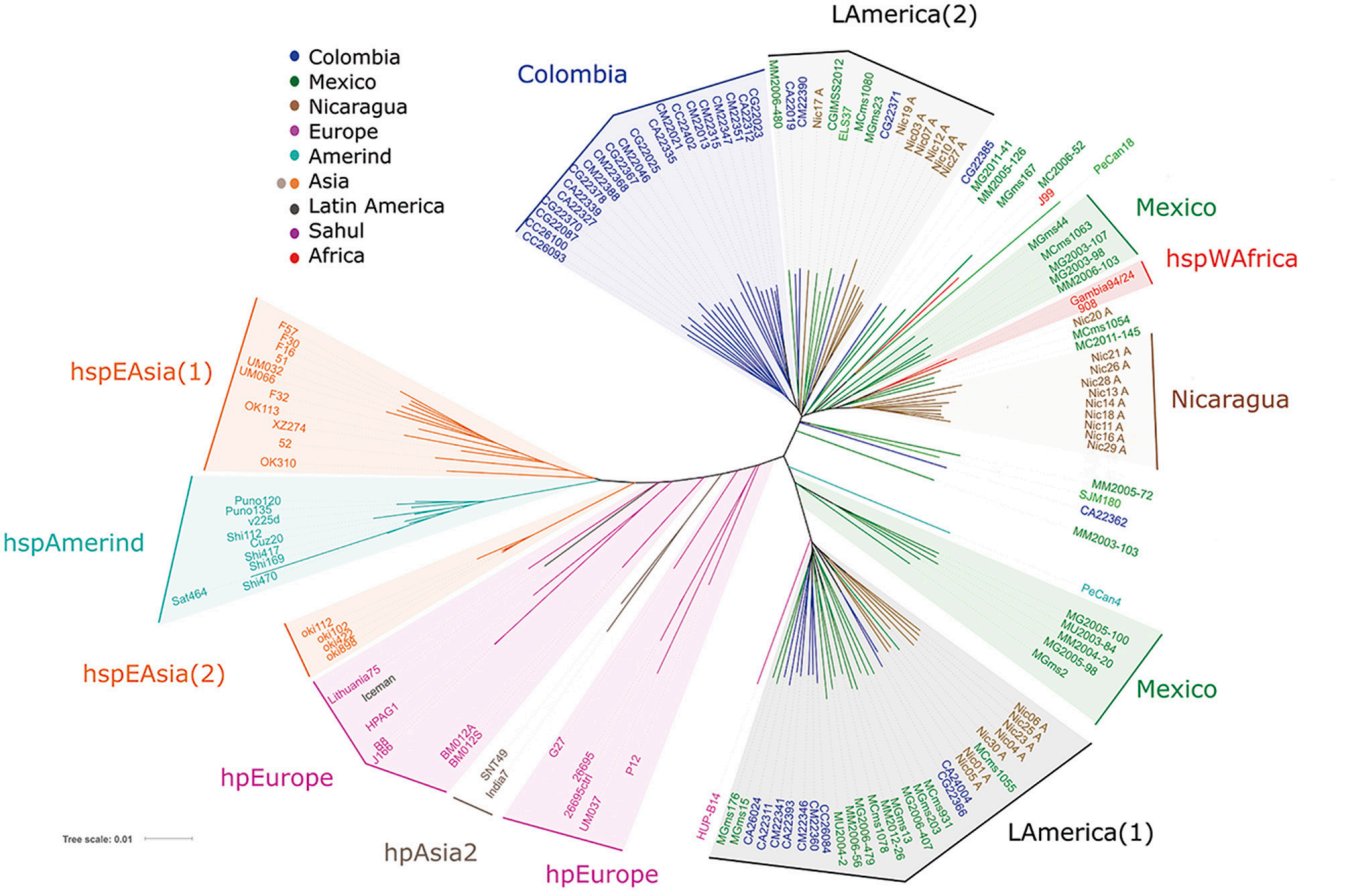
**Phylogenetic analyses of 96 Latin American and 43 worldwide *H. pylori* strains using the sequences of the genes of the *cagPAI* virulence island**. Phylogenetic analyses were done with MEGA 5.02, and distance-based phylogenetic trees were calculated using the T92+G+I model. Phylogenetic trees were edited and annotated with iTol v3.

Based on the above VGF results, we next studied the distribution of *H. pylori* populations restricting the analysis to only the sequences of the *cagPAI* genes. Phylogenetic analyses were done with MEGA 5.02, and distance-based phylogenetic trees were calculated using the T92+G+I model. Phylogenetic trees were edited and annotated with iTol (Interactive Tree Of Life) v3 (Figure 3); hpEurope strains were scattered, and similar to the VGF analysis the LA isolates did not intermingle with them. We observed a very defined LA cluster (LAmerica (1)) composed of Mexican, Colombian and Nicaraguan isolates, and close to this cluster the Spanish HUP-B14. A second LA cluster (LAmerica (2)) was formed with Nicaraguan, Colombian, Mexican and El Salvador strains. Consistent with VGF, a large cluster including only Colombian strains (Colombia group) stood out. There were also other clusters of strains from Nicaragua and from Mexico that grouped close to hspWAfrica. In this *cagPAI* analysis the Iceman strain grouped with hpEurope, similar to VGF results.

### *cagA* gene analyses further confirm the separation of LA strains from hpEurope, hpAsia, and hspAmerind

**Figure 4:**
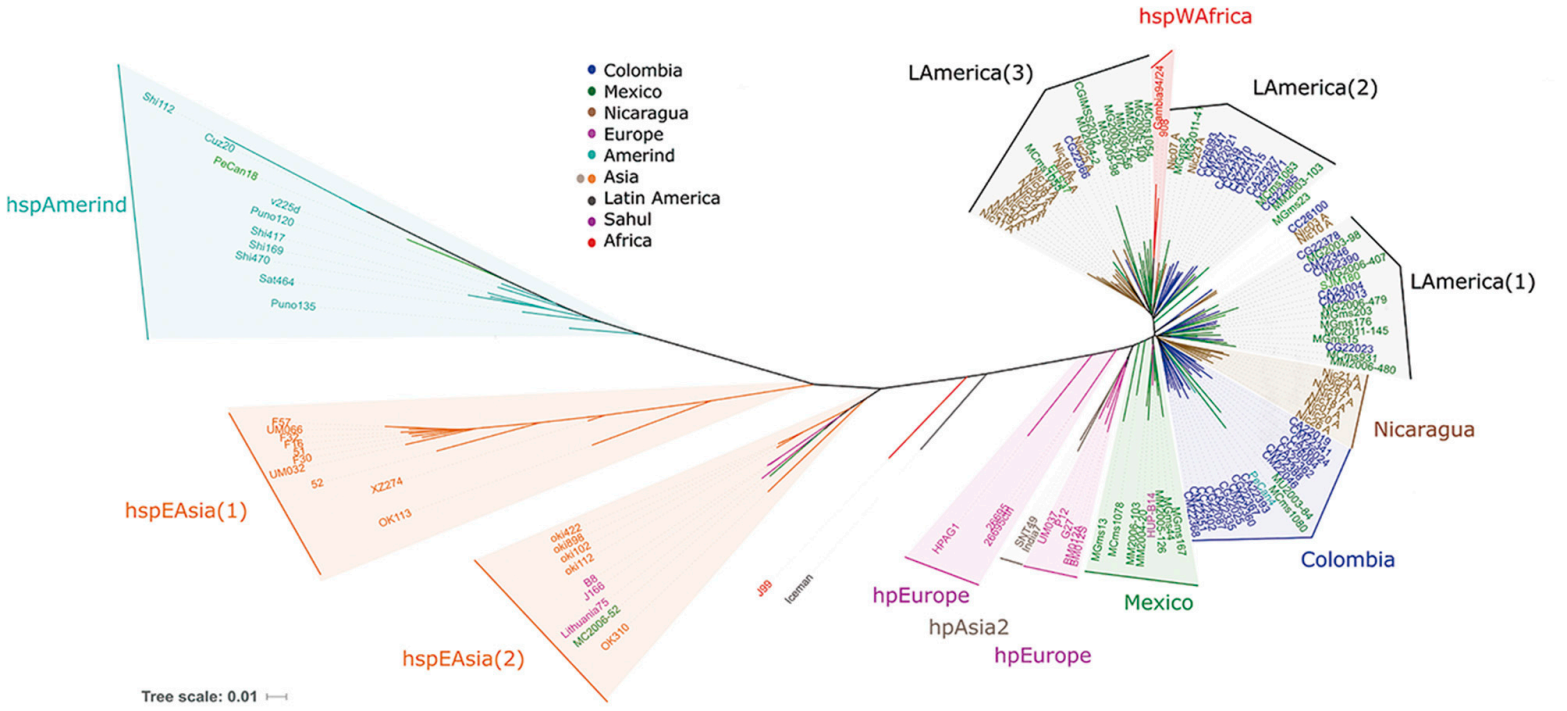
**Phylogenetic analyses of 94 Latin American and 43 worldwide *H. pylori* strains using the sequence of the *cagA* gene**. Phylogenetic analyses were done with MEGA 5.02, and distance-based phylogenetic trees were calculated using the T92+G+I model. Phylogenetic trees were edited and annotated with iTol v3.

Focusing further, we compared the sequence of the *cagA* gene, and similar to previous analyses this split the hspEAsia strains in two, where the hspEasia(2) included European and Mexican isolates (Figure 4). The hpEurope strains were dispersed and none of the LA isolates grouped with them. Furthermore, similar to what was observed with VGF, each LA region formed specific clusters, one large group including mostly Colombian strains (Colombia group, Figure 4), which matched most of the strains included in the Colombia group with all previous analyses (MLST, VGF, and *cagPAI*). We observed two clusters with only Nicaraguan or Mexican isolates, and 3 other including a mix of LA strains, LAmerica(1) with Mexican and Colombian isolates (and SJM180 from Peru), LAmerica(2) with a majority of Colombian isolates, and LAmerica(3) with isolates from Mexico, Nicaragua and El Salvador. The LAmerica(2) and (3) grouped close to hspWAfrica, and we noticed that with the analyses of *cagA*, hspAmerind grouped more distant from the other LA strains than with all previous analyses. With *cagA* gene the *Iceman* sequence localized between hpEurope and hspEAsia.

We also studied *cagL*, and found a different distribution of populations (Figure S2). With this gene hspAmerind and hspEAsia were closer than with VGF, *cagPAI* or *cagA* analyses; and as with MLST, hpEurope intermingled with a number of LA strains. There were also other clusters containing LA isolates of the different regions, but in contrast to previous analyses no clear Colombian, Nicaraguan or Mexican cluster was observed. Of note, the Amerind V225d strain clustered with hspEAsia and strains MM2005-72 from Mexico and PeCan18 from Peru fell within the hspAmerind cluster (Figure S2).

### Phylogenic network analyses show abundant reticulated events between strains

**Figure 5:**
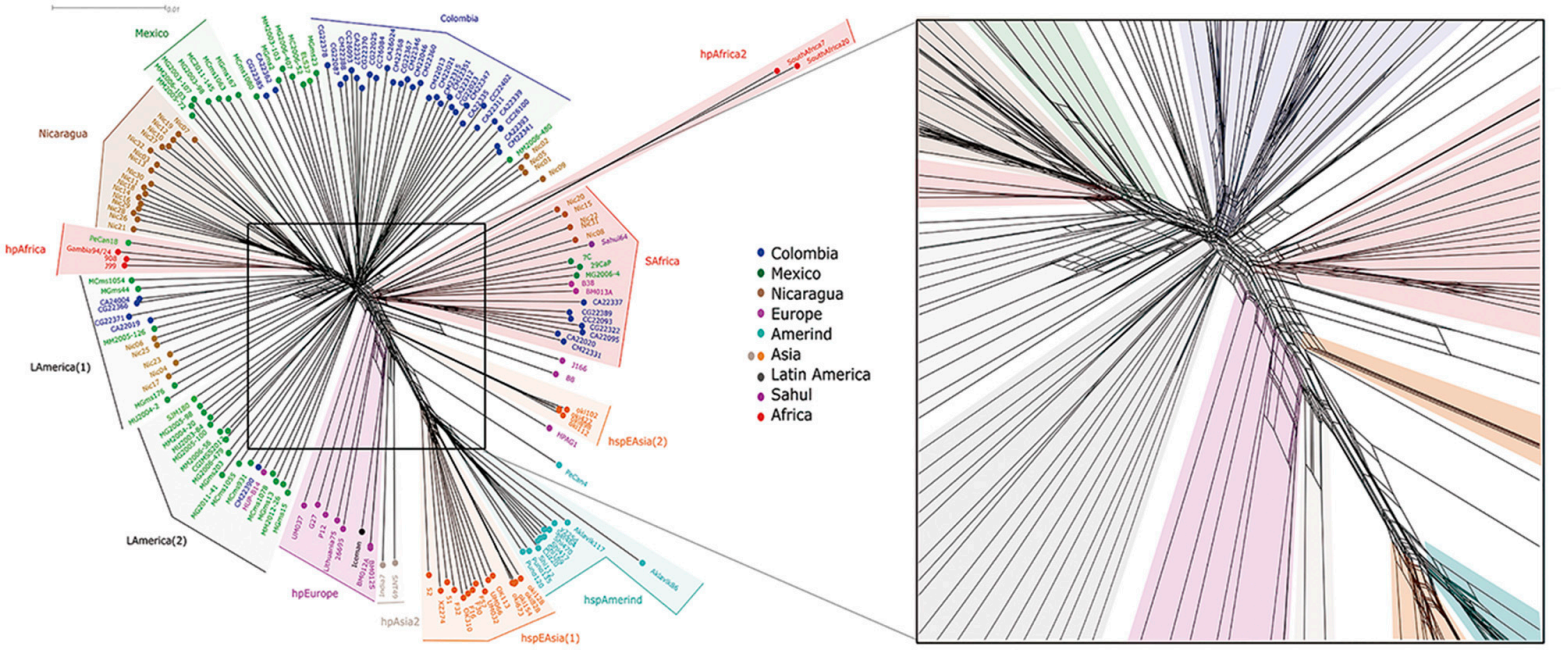
**Phylogenetic network analyses of 113 Latin American and 54 worldwide *H. pylori* strains using whole genome sequences**. To the right is an amplified view of the central part of the figure to better observe the reticulation events among strains.

To study the importance of recombination in the formation of LA groups we ran a phylogenic network analyses (NeighborNet). The results showed numerous reticulated events between most strains, although the most complex reticulated interactions were observed between strains of similar geographic origin (Figure 5). In groups like Colombia or Nicaragua, evidence of both early and recent interactions could be observed, as illustrated by reticulation close to divergent points but also further up along the branches. Evidences of only early interactions were observed between geographically distant groups, such as hpEurope and hspEAsia. The analyses also showed that whereas hpEurope strain HPAG1 presented close interactions with hspEAsia(2), the rest of hpEurope isolates, including the Iceman, had reticulations with hpAsia2 and the LAmerica(2) group.

In accordance with the VGF results, the network analyses showed that hpAfrica2 population shared early reticulations with the SAfrica group. However, when we excluded *cagPAI* from the analyses (Figure S3), all strains in this SAfrica group were relocated, mostly with the hpEurope group, similar to results with VGF (Figure S1). Of interest in both NeighborNets with and without *cagPAI* the PeCan4 strain showed more reticulations with hspAmerind but also early interactions with hspAsia(2), further supporting its hybrid nature.

## Discussion

Our work represents the largest effort to study genomes of *H. pylori* LA strains, and indeed the largest in any human group. In addition, we tested the hypothesis that phylogenetic analyses using whole-genome sequences instead of MLST may lead to the discovery of a distinct population structure, particularly for populations in LA. In our initial analysis with MLST the hpEurope group was rather spread in a number of different clusters and mixed with some of the LA strains, as previously reported (Falush et al., 2003; Thorell et al., 2016). This inconsistent clustering for European strains has been observed previously (Falush et al., 2003) and is the result of the complex history of Europe, populated through many different migration waves. With MLST we found that a fraction of LA strains showed hpEurope ancestry, although other LA clusters grouped separately, already suggesting some adaption to mestizo hosts.

In contrast, the analyses based on whole genomes using VGF resulted in a markedly different population structure, where clusters were observed for each of the three LA countries studied and with no mix with hpEurope. Of note, with VGF most of the Colombian strains grouped in a well-defined cluster, suggesting that this population is separating even from the other LA groups. Also, a large LA cluster including isolates from Mexico, Nicaragua, Colombia, El Salvador and Peru was obtained, indicating the formation of a regional *H. pylori* subpopulation. As hypothesized, analysis including whole genomes resulted in a better resolution of populations, and suggests a structure discordant with that observed with MLST, particularly for the LA mestizo population. Thus, the VGF analysis suggests the existence of LA subpopulations that have evolved to adapt to human groups prevalent in this region. Recently, a whole genome phylogenetic analyses of multi-ethnic Malaysia strains also revealed three major phylogeographic lineages, one clustering with hspEAsia, the other with hpEurope and a third with hspSouthIndia (Kumar et al., 2015), emphasizing the improved resolution with whole genome analyses.

Importantly, the Spanish HUP-B14 strain was also included in the large LA cluster by VGF, which suggests LA strains still retain Spanish ancestry; however, the lack of more Spanish genomes precluded a more informative analysis. Unfortunately, no additional information on this single Spanish strain could be obtained, and it would have been useful to know the region of Spain and if the individual had had any mix with LA people. We should notice that this Spanish strain did not group with hpEurope using VGF, as it did using the more limited MLST analysis, thus making another important difference with whole genome analyses.

Studies have documented that Asian groups populated the Americas through the Bering Strait (Falush et al., 2003), an observation that was confirmed with the genotyping of *H. pylori* isolates from indigenous communities across America, which formed the Amerind group (Kersulyte et al., 2010; Mane et al., 2010; Camorlinga-Ponce et al., 2011). It should be emphasized that our study included urban populations and not indigenous Amerindian groups, and the structure of these mestizo populations is the result of genetic admixture of pre-Columbian American groups with the European colonizers and African slaves. In fact, we observed clusters of Mexican and of Nicaraguan strains that grouped close to hspWAfrica, suggesting that some LA *H. pylori* groups retain a marked African ancestry. Whereas MLST results suggested that hpEurope has displaced the hspAmerind population (Devi et al., 2006; Domínguez-Bello et al., 2008), our VGF analyses rather indicated that LA strains have differentiated into subpopulations evolutionary adapting to human groups of this region. This contrasts with the recent suggestion that in South America, where humans are highly admixed, the human-*H. pylori* relationship may be less likely to reflect co-adaption (Kodaman et al., 2014). It should be noted that these LA groups were more distant from their hspAmerind ancestors and closer to hspWAfrica. A recent study found that Amerind strains are less successful importing than exporting DNA (Yahara et al., 2013), and probably this impaired asymmetric genetic flux might have compromised its ability to adapt to the nascent mestizo populations.

Probably this recent and fast adaption during the last 500 years following the European colonization is the result of high recombination rates, expected to occur in a setting where mixed infections occur frequently. Most LA countries present elevated prevalence of *H. pylori* infection with high transmission rates, which favors mixed infections (González-Valencia et al., 2000; Leal-Herrera et al., 2003; Porras et al., 2013). High rates of recombination within subgroups must be facilitated by the restriction-modification systems; and in accordance with this, a recent study reported that restriction-modification systems in *H. pylori* pose no barrier to homeologous recombination events, as those occurring within our LA subpopulations (Bubendorfer et al., 2016). The adaption of *H. pylori* to its human populations might be an important evolutionary process to reduce acute toxicity and to favor the decades long chronic colonization of the human stomach, but long term consequences of such microevolution need to be further explored. Net effects of reduced pathogenicity may also be host-dependent; sustained chronic infection may lead to increased risk of GC in some hosts, while reduced acute toxicity may result in decreased risk of GC in others. In fact, a recent study in Colombia suggested that infection with a *H. pylori* strain discordant with the genetic background of the patient increase the risk to develop preneoplasic lesions and gastric cancer (Kodaman et al., 2014). It remains to be studied whether this adaption is also occurring in old world populations, particularly in region of Asia where GC mortality is still among the highest in the world.

Interestingly, with VGF some Nicaraguan and Mexican strains, together with two European and one Sahul isolate formed a cluster whose closest ancestors were the hpAfrica2 isolates. However, we noticed that all strains in this cluster lacked the *cagPAI*, and after removing it from the analyses, they all relocate closer to hpEurope, suggesting that *cagPAI* has a strong influence on the genomotype. Notably, some of these strains also grouped close to hpAfrica2 in the MLST analyses, suggesting they do have some similarity with South African strains possibly due to migration of people with South African ancestry to the Americas. Based on the above observations, we were interested in the analyses of the sequence of the *cagPAI* that contains a number of genes encoding proteins with intimate interaction with host proteins, and are consequently under constant positive selection (Delgado-Rosado et al., 2011; Backert et al., 2015). Our analysis of *cagPAI* showed separation of LA from European, Asian and Amerind groups, suggesting a fast adaption of these virulence genes to their human populations, probably as a result of a continuous positive selection facilitated with high recombination rates. In agreement with this, previous publications in LA have reported high positive selection in virulence genes like *cagA, oipA* and *babA* (Torres-Morquecho et al., 2010; Thorell et al., 2016). For the *cagPAI* sequences we also observed clusters of Mexican and Nicaraguan strains grouping close to hspWAfrica, indicating that for some strains even these pathogenicity island genes conserve homology with their Africa ancestors, probably because their human hosts also retain some African ancestry. Although results with VGF and *cagPAI* where similar, some differences were observed; with VGF the number of strains in the “Colombia” clade was larger and only one Latin American group was formed, whereas with *cagPAI* two clearly separated Latin American groups were observed. Grouping of European strains was also better defined with VGF. Some of these differences may arise from the removal of *cagPAI*-negative strains from the latter analysis.

The analyses of the *cagA* gene also resulted in LA clusters, which strongly suggests this is one of the genes that is adapting to the mestizo populations, just as it has been documented for other populations (Kawai et al., 2011). A previous report documented the various mechanism of recombination along the *cagA* gene, which together with positive selection may explain the evolution of the gene in different human groups (Furuta et al., 2011). *cagA* phylogeny contrast with results obtained for *cagL*, where we observed a higher mix of LA and Europe isolates. Whereas *cagA* has been reported to interact with a number of host cell proteins, *cagL* seems to interact only with integrins, and the nature of this interaction might be more conserved among different human groups (Kwok et al., 2007). Still, a recent report suggests that regional diversity in the hypervariable CagL motif (CagLHM) may be related with the variable association of *H. pylori* with gastric cancer in different geographical regions (Gorrell et al., 2016).

*Helicobacter pylori* phylogenetic and phylogeographic studies are challenging because of the complex evolutionary events associated with this bacterium. Several authors have highlighted the high rate of recombination in the genome of this bacterium (Suerbaum et al., 1998; Falush et al., 2001). However, as these events are restricted between strains from hosts living in close proximity, in *H. pylori* recombination is usually restricted geographically, which is also explaining the relatively fast adaption we observed to their LA host groups. To further document these recombination events, we calculated a phylogenic network, derived from distance matrices of whole genomes and obtained a highly reticulated network, which reflects the extent of the recombination effects and other possible horizontal exchange events between *H. pylori* strains. Importantly, we observed that the most abundant and recent reticulation interactions were within LA groups, which suggest that recombination is still driving separation and adaption of *H. pylori* to these mestizo human groups.

We acknowledge that one limitation of the study is that results are partly based on VGF, a method not described previously; however, our findings with this method were confirmed with the analyses of the *cagPAI* genes. In addition, virtual hybridization approaches using word, k-tuple or k-mer techniques (in programs such as Muscle, KAlign, or Mummer) have been commonly used for sequence comparison and phylogenetic analysis, because of their speed and reliability. We should also notice that our group has published works that show the reliability of the phylogenomic classification based in Virtual Genomic Fingerprints. (Hueman et al., 2011, 2015; Reyes-Prieto et al., 2011), and VAMPhyRE is an improved version of these earlier methods. Finally, a detailed description of the method will be submitted for publication shortly, including validation studies using both bacteria and viruses (Mendez et al., manuscript in preparation). Another limitation of our study is that we genotyped *H. pylori*, but did not characterize the patients; in this sense, it is well documented that Latin American populations are the result of extensive admixture of Native Americans, Europeans, and Africans, termed as mestizo population; studies have shown predominant European and Native American ancestry with low level of African ancestry (Wang et al., 2008).

In conclusion, this work represents the largest effort to study LA *H. pylori* strains, sequencing a collection of over 100 whole genomes. The study shows that the analyses of whole genomes improved the definition of *H. pylori* populations, particularly in LA. The adaption of *H. pylori* to regional human groups suggests that in the new world, *H. pylori* has evolved to fit LA subpopulation already 500 years after the Spanish colonization. This ongoing adaption is evident also with the analyses of *cagPAI* virulence island genes. This fast co-evolution is must probably due to high recombination within populations in a setting of highly frequent transmission that favors the occurrence of mixed infections. This fast co-evolution may account for regional variability in gastric cancer risk within LA populations.

This Whole Genome Shotgun project has been deposited at DDBJ/ENA/GenBank as follow, for the Mexican strains under the bioproject PRJNA338771 and accession numbers SAMN05569559-SAMN05569592; whereas for the Colombian strains under the bioproject PRJNA352848 and accession numbers SAMN06187417-SAMN06187458.

## Author contributions

JT designed the study, worked on the analyses of data, and wrote the manuscript; ZM participated in the bioinformatics analysis; AM participated in analysis of data and writing the manuscript; IK participated in the design of the study and analysis of data; MB participated in the coordination of the clinical work and in the recruitment of cases from Colombia; CR participated in the design of the study and analysis of data; KT contributed with the analysis of the Nicaraguan genomes and with writing of the manuscript; RT participated in the phylogenic network analysis; FA participated in analysis of data and drafting the manuscript; MC participated in the coordination of the clinical work and in the recruitment of cases from Mexico and FC contributed with the genome sequence of Mexican and Colombian strains.

## Funding

This work was supported by “Consejo Nacional de Ciencia y Tecnología,” Mexico City, Mexico [406852 to ZM], the “Programa Institucional de Formación de Investigadores” Instituto Politécnico Nacional [20162112 to ZM] and the National Institutes of Health, United States [5R21CA182822 to IK].

### Conflict of interest statement

The authors declare that the research was conducted in the absence of any commercial or financial relationships that could be construed as a potential conflict of interest.

## Footnotes

^1^https://www.cia.gov/library/publications/the-world-factbook/geos/nu.html
