# Supplementary material for: Whole Genome Sequence and Phylogenetic Analysis Show Helicobacter pylori Strains from Latin America Have Followed a Unique Evolution Pathway
Source: Front Cell Infect Microbiol. 2017 Feb 28;7:50. doi: 10.3389/fcimb.2017.00050 (PMC5328995; doi:10.3389/fcimb.2017.00050)
Supplement: Supplementary file 1 [file Table1.PDF]

Table *S*1. Origin and GenBank accession number of genomes downloaded for this study.

Strain	Region	GenBank number
26695	hpEurope	NC_000915.1
HPAG1	hpEurope	NC_008086.1
G27	hpEurope	NC_011333.1
P12	hpEurope	NC_011498.1
B38	hpEurope	NC_012973.1
Lithuania75	hpEurope	NC_017362.1
HUP-B14	hpEurope	NC_017733.1
B8	hpEurope	NC_014256.1
J166	hpEurope	NZ_CP007603.1
BM012A	hpEurope	NC_022886.1
BM012S	hpEurope	NC_022911.1
BM013A	hpEurope	NZ_CP007604.1
UM037	hpEurope	NC_021217.3
Aklavik117	hspAmerind	NC_019560.1
Aklavik86	hspAmerind	NC_019563.1
Shi470	hspAmerind	NC_010698.2
v225d	hspAmerind	NC_017355.1
PeCan4	hspAmerind	NC_014555.1
Cuz20	hspAmerind	NC_017358.1
Sat464	hspAmerind	NC_017359.1
Puno120	hspAmerind	NC_017378.1
Puno135	hspAmerind	NC_017379.1

Shi417	hspAmerind	NC_017739.1
Shi169	hspAmerind	NC_017740.1
Shi112	hspAmerind	NC_017741.1
J99	hpAfrica1	NC_000921.1
Gambia94/24	hpAfrica1	NC_017371.1
SouthAfrica7	Africa	NC_017361.1
SouthAfrica20	Africa	NC_022130.1
51	hpEastAsia	NC_017382.1
52	hpEastAsia	NC_017354.1
XZ274	hpEastAsia	NC_017926.1
F16	hpEastAsia	NC_017368.1
F30	hpEastAsia	NC_017365.1
F32	hpEastAsia	NC_017366.1
F57	hpEastAsia	NC_017367.1
OK113	hpEastAsia	NC_020508.1
OK310	hpEastAsia	NC_020509.1
UM032	hpEastAsia	NC_021215.3
UM066	hpEastAsia	NC_021218.3
oki102	hpEastAsia	NZ_CP006820.1
oki112	hpEastAsia	NZ_CP006821.1
oki128	hpEastAsia	NZ_CP006822.1
oki154	hpEastAsia	NZ_CP006823.1
oki422	hpEastAsia	NZ_CP006824.1
oki673	hpEastAsia	NZ_CP006825.1

oki828	**hpEastAsia**	NZ_CP006826.1
oki898	**hpEastAsia**	NZ_CP006827.1
India7	**hpAsia2**	NC_017372.1
SNT49	**hpAsia2**	NC_017376.1
29CaP	**Mexico**	NZ_CP012907.1
7C	**Mexico**	NZ_CP012905.1
CGIMSS2012	**Mexico**	GCA_000472305.1
SJM180	**Latinoamerican**	NC_014560.1
PeCan18	**Latinoamerican**	NC_017742.1
ELS37	**Latinoamerican**	NC_017063.1
Sahul64	**hpSahul**	PRJNA172821
Iceman	**-**	ERP012908
Sheeba (*H. Acinonychis*)	**-**	NC_008229.1
