# Supplementary figures and images for: Whole Genome Sequence and Phylogenetic Analysis Show Helicobacter pylori Strains from Latin America Have Followed a Unique Evolution Pathway
Source: Front Cell Infect Microbiol. 2017 Feb 28;7:50. doi: 10.3389/fcimb.2017.00050 (PMC5328995; doi:10.3389/fcimb.2017.00050)

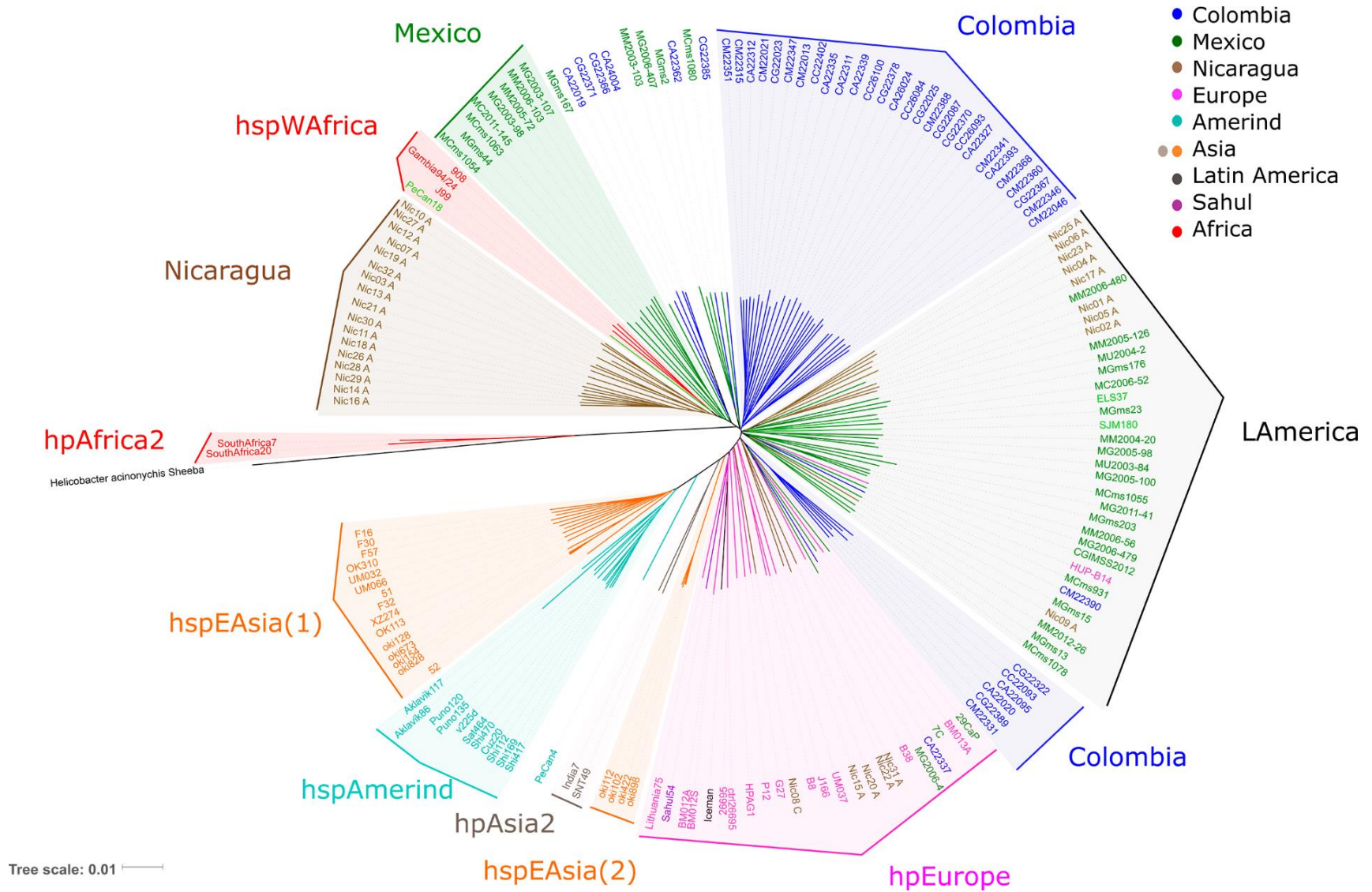

**Figure S1.** Virtual genome fingerprint analyses without the *cagPAI* island.

Supplement: Supplementary file 2 [file Image1.PDF]

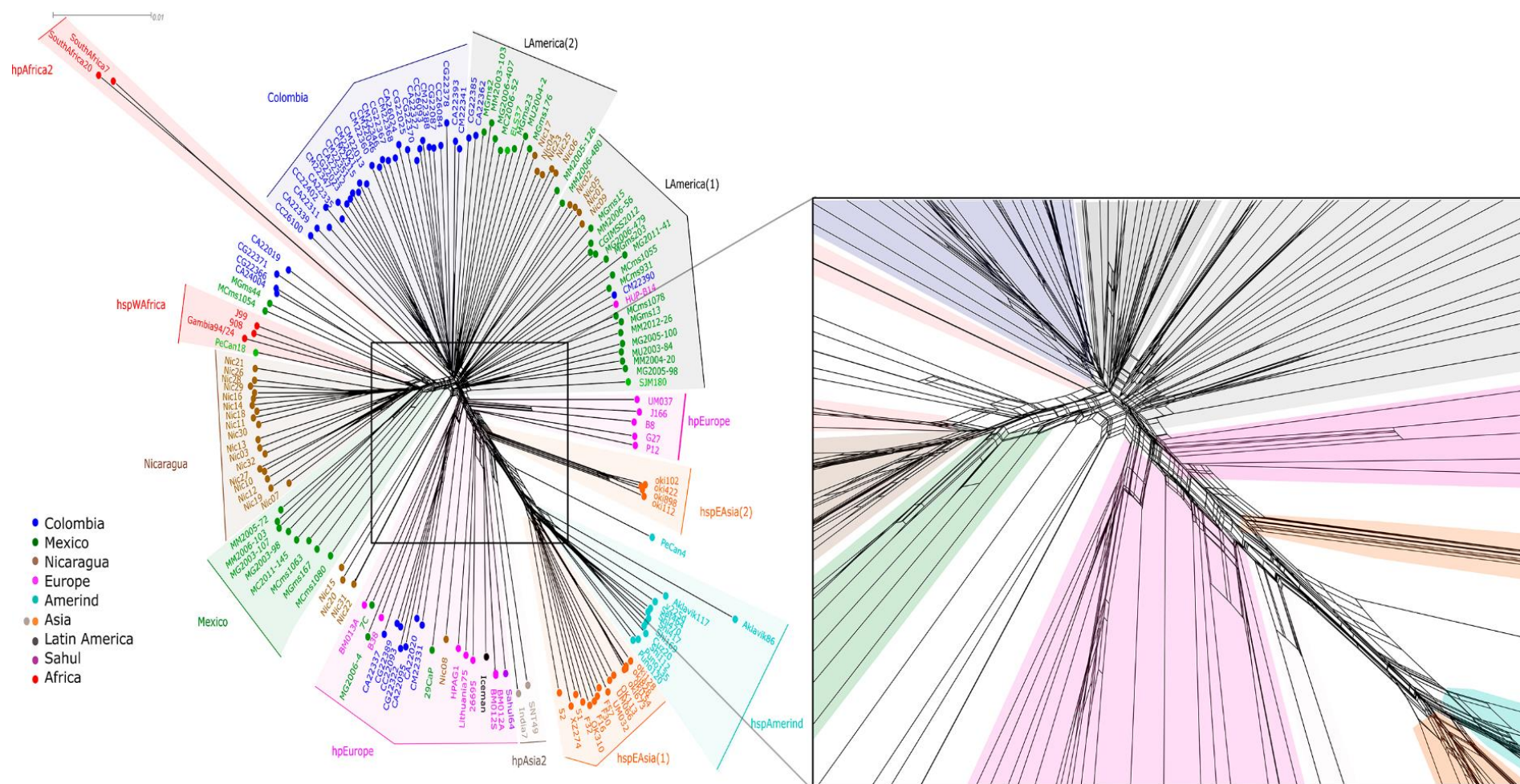

**Figure S3.** Phylogenetic network analyses without the *cagPAI* island.

Supplement: Supplementary file 4 [file Image3.PDF]
