# Supplementary material for: Whole Genome Sequence and Phylogenetic Analysis Show Helicobacter pylori Strains from Latin America Have Followed a Unique Evolution Pathway
Source: Front Cell Infect Microbiol. 2017 Feb 28;7:50. doi: 10.3389/fcimb.2017.00050 (PMC5328995; doi:10.3389/fcimb.2017.00050)
Supplement: Supplementary file 3 [file Image2.PDF]

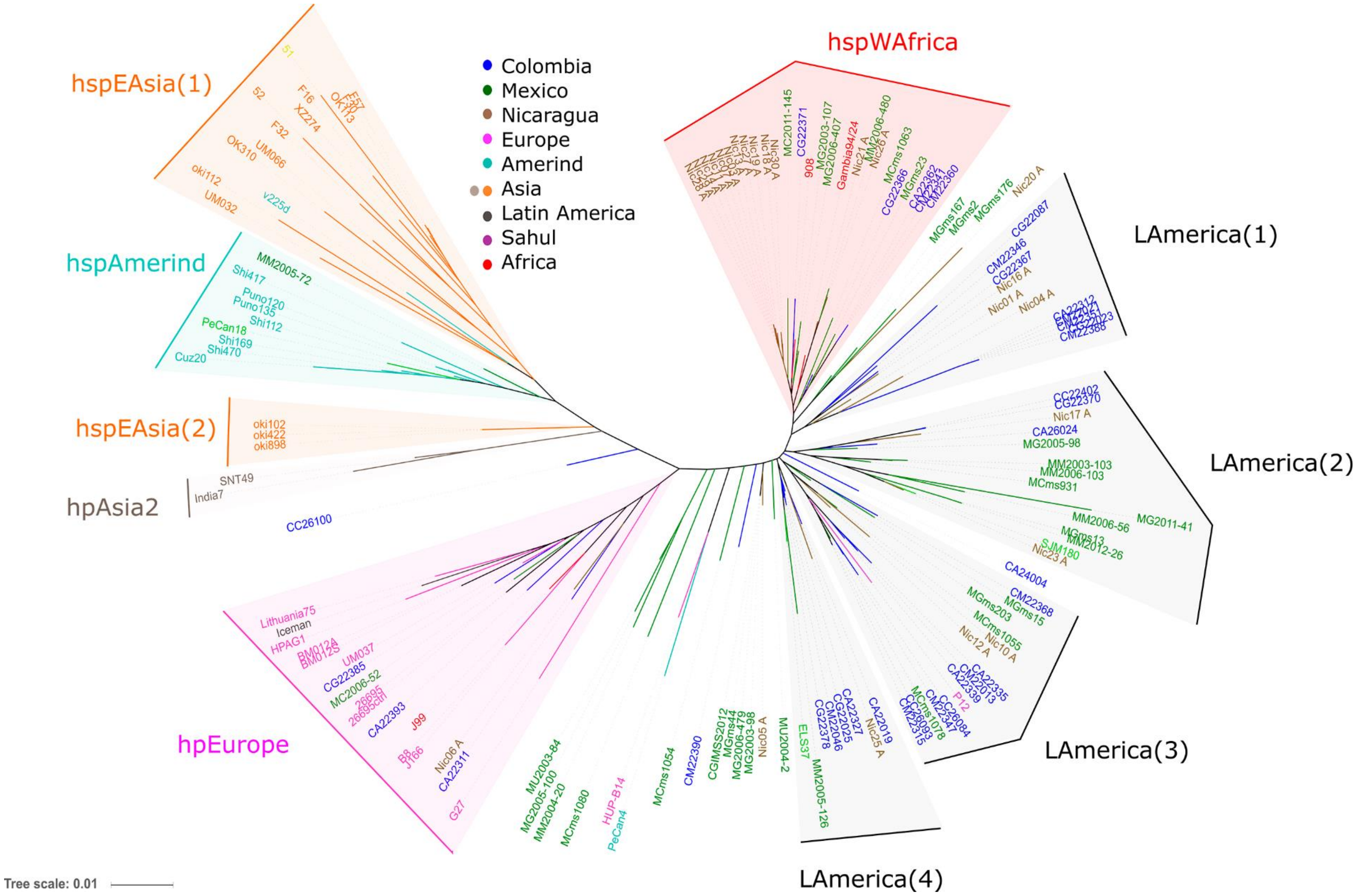

Figure *S2*. Phylogenetic analyses using the *cagL* gene.
